# Historical and Contemporary Debates in Schlemm’s Canal-Based MIGS

**DOI:** 10.3390/jcm13164882

**Published:** 2024-08-19

**Authors:** Etsuo Chihara, Teruhiko Hamanaka

**Affiliations:** 1 Sensho-Kai Eye Institute, Minamiyama 50-1, Iseda, Kyoto 611-0043, Japan; 2 Japanese Red Cross Medical Center, 4-1-22 Hiroo, Shibuya-ku, Tokyo 150-8935, Japan; hamanaka.teruhiko@gmail.com

**Keywords:** minimally invasive glaucoma surgery (MIGS), Schlemm’s canal opening surgery, trabeculotomy, trabeculectomy, canaloplasty, Kahook dual blade, trabectome, Tanito micro-hook, surgical success, history, T hook

## Abstract

Glaucoma is one of the primary causes of blindness worldwide. Canal opening surgery, a type of minimally invasive glaucoma surgery (MIGS) applied in cases of mild to moderate glaucoma, has gained increasing popularity in recent years due to its efficacy in reducing the intraocular pressure, its safety profile, the simplicity of its technique, and the reduced likelihood of compromised vision. Nevertheless, the existing body of histopathological studies remains insufficient for a comprehensive understanding of post-surgical wound healing. Consequently, debates persist among researchers regarding the mechanism through which Schlemm’s canal opening surgery reduces the intraocular pressure, as well as the surgical techniques that may impact the outcomes and the factors influencing surgical success. As the history of MIGS is relatively short and lacks sufficient systemic reviews or meta-analyses evaluating the influence of individual factors, this review was conducted to illuminate the disparities in researchers’ opinions at the current stage of research.

## 1. Introduction

Ideally, glaucoma surgery must be safe and effective in reducing the intraocular pressure and maintaining the patient’s visual function. Filtering surgeries are effective in reducing the IOP; however, the deterioration of post-surgical visual acuity is common [1,2], and many problems occur, such as post-surgical hypotension, bleb leaks, infection, and others. Because of these problems and the complexity of postoperative care, there has been a shift from filtering surgery to non-filtering surgery. Canal opening surgery (Schlemm’s canal-based MIGS) is a type of minimally invasive glaucoma surgery (MIGS) that has gained popularity in recent years. However, the history of MIGS is short, and controversies remain unresolved. This study provides an overview of the history and clinical controversies of canal opening surgery.

## 2. History of Canal Opening Surgery

### 2.1. Evolution of Trabeculotomy Ab Externo

As early as 1873, it was established that the trabecular meshwork is the main site of aqueous humor outflow resistance in glaucoma eyes [3]. In 1954, Barany and Scotchbrook found that the injection of testicular hyaluronidase significantly increased the aqueous outflow [4]. 

In 1947, Barkan hypothesized that incising the trabecular meshwork, which was postulated as the primary site of outflow resistance, could reduce the intraocular pressure [5]. He introduced the concept of goniotomy. This technique offered great relief for refractory congenital glaucoma patients [6]. However, at that time, the absence of appropriate surgical microscopes posed a significant challenge in accessing and incising the trabecular meshwork from inside the eye. Consequently, goniotomy was primarily indicated for congenital glaucoma, which was associated with poor outcomes after filtration surgery. 

Subsequently, in England, Smith achieved success by opening Schlemm’s canal through a blunt incision in the trabecular meshwork [7]. This was accomplished by inserting a thread into Schlemm’s canal and pulling it; this technique was also reported by Burian around the same period [8]. 

Nine years later, Smith provided an update on his continued efforts. He said, “I have been performing Nylon filament trabeculotomy for seven years, but I’ve been holding back from writing papers in the meantime. The reason for this was that I was not sure whether the surgery was really benefiting the patient”. Subsequently, he detailed the outcomes. The outcomes of 26 cases of suture trabeculotomy and 27 cases of filtering surgery (nine irido-encleisis, eight Scheie, six trephine, and four Stallard surgeries) were compared. The results indicated a more favorable decrease in intraocular pressure with suture trabeculotomy [9]. 

Trabeculotomy using a thread was technically challenging in the era before the development of surgical microscopes. Subsequently, trabeculotomy using metal probes was invented in Germany, and it became a widely adopted technique [10,11,12]. 

### 2.2. Trabeculotomy Ab Externo: A Time of Hardship 

Outflow surgery was a prominent topic at that time. A comprehensive exploration of the histopathological aspects of outflow surgery took place during the 76th American Academy of Ophthalmology (AAO) meeting (Symposium: Microsurgery of the outflow channels), held in Las Vegas from 20 to 24 September 1971. The symposium presented the results of histopathological studies on trabeculectomy, sinusotomy, and trabeculotomy.

During the same symposium, it was demonstrated that aqueous outflow is a passive process and that the outflow ability (µL/min/mmHg) is enhanced by trabeculotomy. However, the opening of Schlemm’s canal in normal monkey eyes closed within 28 weeks after surgery [13], and the ability to enhance the outflow diminished over time. Grant suggested that leaks from the flap and inadvertent cyclodialysis may lead to post-surgical hypotension [14]. Trabeculotomy ab externo was reported as a challenging procedure that carried the risk of the misinsertion of the probe outside Schlemm’s canal [15]. 

Despite the AAO’s critical debate on the promotion of trabeculotomy, the publication of studies recommending trabeculotomy ab externo did not stop [16,17,18]. Dannheim subsequently reported a stable reduction in intraocular pressure for 4 years following trabeculotomy surgery [19] and reported the facilitation of aqueous outflow using tonography [20]. Mackensen noted that “even though effects of trabeculotomy in pressure-reducing is inferior to that of fistulizing surgery, authors prefer trabeculotomy because this preserves the unaffected meshwork” [21]. 

Despite the favorable clinical reports, the negative impact of the experimental studies, which demonstrated the early closure of the trabecular opening after trabeculotomy and no improvement in the rate of aqueous humor outflow in monkeys, was substantial [19,22,23]. Due to these negative results, the validity of trabeculotomy ab externo as a treatment for adult-onset primary open-angle glaucoma (POAG) was questioned at that time. 

### 2.3. Trabeculotomy Ab Externo: Exploring a Means to Survive as a Treatment for Congenital Glaucoma

In a prospective study, Luntz et al. treated 19 cases of adult-onset glaucoma with a mean intraocular pressure (IOP) of 41 mmHg using trabeculotomy ab externo, achieving a success rate of 70%. In contrast, trabeculotomy in 19 cases of congenital glaucoma with a mean IOP of 34 mmHg achieved success in 18 out of 19 eyes (95%). The authors concluded that trabeculotomy was a procedure for congenital glaucoma and not for adult-onset POAG [24]. 

Subsequently, trabeculotomy ab externo gained recognition as a viable option for the treatment of congenital glaucoma and infantile glaucoma. It was acknowledged as being equally effective as goniotomy, solidifying its status as a treatment for children at that time. 

The disparity in efficacy between adult-onset POAG, which showed poor results, and infantile or congenital glaucoma, which exhibited positive outcomes, was attributed to the presence of rich elastic fibers in the infantile angle, which could increase the gaping of elastic tissue in the angle structure [25].

## 3. Historical Controversy (Part I): Clinical Aspects

### 3.1. Goniotomy vs. Trabeculotomy Ab Externo; Which Is the Optimal Approach for Congenital Glaucoma? 

As previously mentioned, trabeculotomy ab externo gained recognition as a viable treatment for congenital glaucoma in Europe and the United States. As a next step, the debate on the comparative merits of trabeculotomy and conventional goniotomy came to a focal point. 

Some of the authorities argued that congenital glaucoma patients should undergo at least one goniotomy before considering trabeculotomy [26].

McPherson compared the surgical outcomes for congenital glaucoma between trabeculotomy using a modified Harms Trabectome and goniotomy. The study concluded that the outcomes of trabeculotomy ab externo yielded superior results compared to traditional goniotomy. Additionally, the success rate of trabeculotomy was higher in congenital glaucoma cases than in adolescent glaucoma cases [27,28,29].

Rothkoff introduced a modified surgical technique that excised the deeper flap of the doubled flap and reported improved outcomes [30]. 

Glaucoma authorities of this era also reported that trabeculotomy was the recommended surgical approach for congenital and developmental glaucoma. Quigley and Anderson reported that trabeculotomy and goniotomy exhibited comparable performance, with fewer complications in the case of childhood glaucoma. Furthermore, they documented that trabeculotomy ab externo had an advantage when dealing with cloudy cornea. Both researchers also highlighted the correlation between a child’s age and the success of the outcomes [31,32]. 

Subsequently, particularly in the United States, trabeculotomy gained recognition as a viable treatment option for congenital and juvenile glaucoma. At the same time, it was recognized that the outcomes deteriorated as the patient aged. This concept had widespread acceptance both within and outside the USA [33]. 

Before the widespread adoption of internal trabeculotomy (trabeculectomy), external trabeculotomy was frequently employed for congenital glaucoma. However, as internal trabeculotomy gained popularity, there was a shift from external to internal trabeculotomy, and it is becoming the preferred method over external trabeculotomy [34,35,36,37,38,39,40,41,42,43].

### 3.2. Historical Controversy (Part II): Is Trabeculotomy Ab Externo Effective for Adult POAG? 

Before the introduction of the Trabectome in 2005, Lamers [18], Dannheim et al. [19], and Nagata et al. [44,45] reported the long-term success of trabeculotomy ab externo in controlling the intraocular pressure in adult-onset POAG.

In the “Tübingen Study”, Weder and others compared the surgical outcomes between trabeculotomy ab externo and goniotrephination. They concluded that trabeculotomy should be considered as an alternative operation for elderly patients with glaucoma simplex to avoid hypotonia. Their study revealed that two thirds of 80 POAG cases were controlled without medication, and 89% achieved qualified success [46]. In 1999, Quaranta and others reported that trabeculotomy could be applied in adult-onset POAG. They compared the outcomes between trabeculotomy and trabeculectomy augmented with mitomycin C in adult POAG, finding no statistically significant difference in the IOP from the 6th to the 24th postoperative month between the two groups [47]. 

Before 2004, the clinical effectiveness of external trabeculotomy was mainly highlighted in Japan, with multiple publications supporting its effectiveness in adult POAG and PEG [48,49,50,51,52,53,54,55,56,57,58]. A user-friendly probe for trabeculotomy, invented by Nagata, might have facilitated the widespread adoption of this technique in the country. In 2002, Tanito et al. utilized Cox multivariate analysis and reported that advanced age is a favorable prognostic factor for the successful control of the IOP through trabeculotomy ab externo. Their findings also demonstrated a good post-surgical visual acuity prognosis, highlighting that elderly POAG patients with a concomitant cataract are well suited for combined phacoemulsification aspiration, the implantation of an IOL, and trabeculotomy ab externo surgery [59]. At that time, the efficacy of trabeculotomy ab externo was widely accepted and it was adopted as a standard treatment for mild to moderate POAG in Japan. Around 2008, trabeculotomy ab externo accounted for 38% of the total number of glaucoma surgeries performed in Japan [60]. 

In spite of these reports, canal opening surgery was not recognized as an effective treatment for POAG in adults globally until 2005. The shift towards canal opening surgery in the United States began to emerge in 2005. In 2005, the invention of the Trabectome^®^ by Minckler marked the initiation of internal trabeculotomy, which was later named MIGS [25,61]. 

In a review article, Godfrey and others introduced canal surgeries such as the Trabectome procedure, canaloplasty, and trabeculotomy ab externo as effective treatments for adult glaucoma. They noted that “in other parts of the world, where canal surgery is more popular, trabeculotomy remains an option to filtering surgery for adult and juvenile glaucoma [62]”. 

Following the publication of this review article, it appears that many glaucoma surgeons have embraced the view that canal opening is an effective treatment for adult patients with POAG or PEG. 

## 4. Historical Controversy (Part III): Histological Aspects

### Debate in Histological Findings after Trabeculotomy: Difference in Tissue Response between Monkeys and Humans

In an earlier study, Dannheim and colleagues investigated the post-surgical regenerative closure of Schlemm’s canal opening in monkey eyes following external trabeculotomy using a metal probe. They observed cellular regeneration in 17 healthy monkeys and noted that this regeneration was initiated between 6 and 8 weeks after the opening of the trabecular meshwork. The trabecular opening closed within 28 weeks due to the regenerated trabecular lamellae and trabecular endothelium, ultimately forming a dense scar [23]. In their study, the Schlemm’s canal opening was filled with fibrous tissue, and the IOP did not decrease in healthy monkey eyes. The closure of the Schlemm’s canal opening in their study might have been considered a potential cause of the poor reduction in IOP. 

Ito and colleagues [63] conducted an electron microscope study of the regeneration of the trabecular meshwork following trabeculotomy ab externo in 10 monkey eyes. Their research revealed that the regeneration of the trabecular meshwork begins with the regeneration of the corneo-scleral meshwork and endothelium, eventually progressing to the regeneration of the uveal meshwork. After one year, the defects were almost completely repaired by newly formed trabecular tissue, which closely resembled normal trabecular tissue. Notably, there was no direct communication between Schlemm’s canal and the anterior chamber at the one-year mark. 

**Figure 1:**
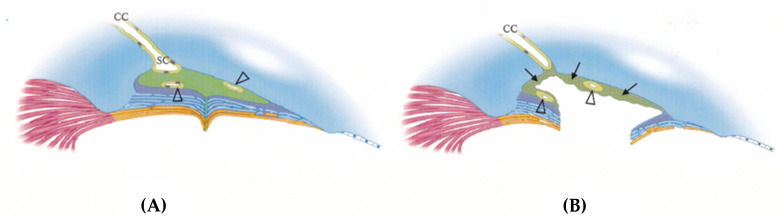
A schematic drawing depicting the histopathology of human eyes following an unsuccessful trabeculotomy [66]. Surgical opening of the trabecular meshwork resulted in closure in 14 of 31 eyes (**A**) and it remained open in 10 of 31 eyes (**B**) to the anterior chamber six years (ranging from 3.4 to 22.6 years) after external trabeculotomy. These specimens were obtained during subsequent trabeculectomy after the initial trabeculotomy ab externo. In 10 eyes, in which Schlemm’s canal (SC) remained open to the anterior chamber, the inner wall of the SC was lined with extended Schlemm’s canal endothelium and/or fibrous proliferation. In 14 eyes with a closed SC, the trabecular meshwork showed sign of fibrous degeneration. Additionally, the intracanalicular space might be filled with fibrous proliferation, leading to the shrinkage of the SC space. In four eyes, the trabecular meshwork was covered by Schlemm’s canal endothelial cells, and, in three eyes, peripheral anterior synechiae covered the trabecular meshwork. Open triangles indicate lumens surrounded by the extended endothelium of Schlemm’s canal. Arrows point to fibrotic tissue surrounding the expanded Schlemm’s canal. (Copyright License #5796400157661 by Exp. Eye Res. Elsevier).

In contrast to the reports by Dannheim [23] and Ito [63], Hamanaka and colleagues conducted a study of the histopathological changes in the trabecular meshwork (TM) and Schlemm’s canal of human eyes where trabeculotomy ab externo had been unsuccessful [64,65,66]. They reported that, in 10 out of 31 eyes, Schlemm’s canal remained open to the anterior chamber even six years after trabeculotomy ab externo [65,66]. In these cases, the inner wall of Schlemm’s canal was either covered or filled with fibrous tissue or the regenerated endothelium of Schlemm’s canal (Figure 1). This finding aligns with the histopathology observed in human eyes by d’Epiney [67], who used electron microscopy to investigate the histopathology of the angle following trabeculotomy in two human eyes with congenital glaucoma. They found that Schlemm’s canal contained abundant erythrocytes, and the inner wall of the canal exhibited an encroached endothelium. In the trabecular area, the trabecular structures were lost and were replaced by cell- and fiber-rich tissue. 

These findings indicate potential differences between monkey eyes and human eyes. 

Notably, the presence of an endothelial layer covering the uveal meshwork was observed in individuals who underwent laser trabeculoplasty; these findings may align with post-trabeculotomy observations [68].

It is crucial to emphasize a key distinction between monkey eyes and human eyes. In monkey eyes, the Schlemm’s canal opening closes within 28 weeks, whereas this phenomenon does not occur in the human eye. 

Regarding the functional aspects of the trabecular meshwork, the outflow after trabeculotomy did not change significantly in “normal” monkey eyes [22]. In contrast, clinical studies in humans have indicated that the reduction in IOP persists for a long period of time [19,48,49,50,51,52,53,54,55,56,57,58]. 

From a clinical perspective, when eyes are examined using gonioscopy following canal opening surgery, it is common to observe a rough-surfaced, pigmented, membrane-like tissue that covers the inner surface of Schlemm’s canal following internal trabeculotomy and internal trabeculectomy. Despite the formation of this membrane on the inner surface of Schlemm’s canal, delayed-onset hyphema may occur after internal trabeculotomy or trabeculectomy. This finding suggests direct communication between the anterior chamber and collector channel [69,70,71,72,73]. 

The enhancement of the episcleral venous fluid wave (EVFW) in the aqueous vein serves as a sign of activated aqueous outflow following canal surgery [74]. 

In normal eyes, the reported number of collector channels (CCs) is approximately 80 [75]. The inferior half of the CCs drain 87% and the inferior nasal CCs drain 56% of the total outflow [76]. If the orifice of the CC becomes sealed by regenerated endothelium and/or fibrous tissue, there is potential for an elevation in the IOP (Figure 1). 

Currently, we lack sufficient information regarding the degree or number of closed CCs that is crucial to impair the normal aqueous outflow. In a previous report, it was suggested that only one or two active aqueous veins may be sufficient to drain enough aqueous humor from the eye [77,78]. The regeneration and fibrosis of the TM tissue, along with the sealing of CCs by fibrous tissue or Schlemm’s canal endothelium, may lead to a gradual increase in the post-surgical IOP [79].

It is important to note that any type of surgical intervention can impact the morphology of the trabecular meshwork [68,80].

## 5. Evolution of Internal Trabeculotomy in Minimally Invasive Glaucoma Surgery (MIGS)

### 5.1. Evolution of 360-Degree Suture Trabeculotomy (Complete Trabeculotomy)

Suture trabeculotomy was first reported by Smith in 1960 [7]. With this technique, the trabecular meshwork is not excised. A nylon thread is inserted into Schlemm’s canal and pulled to open the trabecular meshwork by blunt trauma. In 1987, Lynn and Fellman gave an oral presentation on a 360-degree suture trabeculotomy. Subsequently, in 1987, Beck et al. conducted a study on the outcomes in 26 eyes of 15 patients [81]. In 2012, Chin and others reported a positive 2-year outcome of 360-degree suture trabeculotomy using a 5-0 nylon suture from outside the eye [82]. 

Two years later, Grover and others introduced a technique named gonioscopy-assisted transluminal trabeculotomy (GATT), whereby a microcatheter or suture is used for a complete 360-degree trabeculotomy from inside the eye. They identified the “trabecular shelf” as a sign of an opened Schlemm’s canal [83].

In 2014, Sato and others reported a modified technique involving the simple insertion of a nylon thread without the need for any special instruments [84].

The GATT technique encountered challenges during its initial introduction [85], and it was necessary to undergo a learning curve to acquire proficiency; however, it is now widely accepted as a viable option for the treatment of both adult POAG and childhood glaucoma. Many reports have attested to the long-term success of GATT in effectively controlling the IOP [86,87,88,89,90,91,92,93,94,95,96,97,98,99,100,101,102]. Recently, the combination of internal trabeculotomy and canaloplasty has been reported [103,104,105,106]. It will be interesting to see whether the combined approach of canaloplasty and trabeculotomy yields improved outcomes.

The histopathology of the angle after 360-degree suture trabeculotomy was reported by Hamanaka and others. In their study of 11 cases with unsuccessful IOP control, Schlemm’s canal remained open in 9 out of 11 eyes at 4 years after surgery. The identified cause of failure was the fibrous proliferation and elongation of the Schlemm’s canal endothelium (SCE), which was not associated with the closure of the orifice of the Schlemm’s canal opening [66]. 

There is an ongoing debate regarding whether the outcomes of a 360-degree circumferential trabecular meshwork opening surpass those achieved with a canal opening in the range of 90–150 degrees (see Section 6.2 for further details). 

### 5.2. Evolution of Trabectome^®^ and Kahook Dual Blade

The Trabectome^®^, which was introduced by Minckler and others in 2005, stands as the pioneering device for the ablation of the trabecular meshwork from inside, leading to the evolution of MIGS [25,61]. They introduced a hand piece featuring a 19-gauge infusion sleeve and a 25-gauge aspiration port with a moderately sharp triangular tip. The inner wall of the trabecular meshwork was ablated in a manner similar to bipolar cautery [25]. With this technique, the conjunctiva is completely preserved, and, in the case of failure, it permits subsequent filtering surgeries, such as trabeculectomy and tube shunt surgery. 

As a next-generation internal trabeculectomy device, the Kahook dual blade was invented; it enabled the excision of the trabecular meshwork through the use of a dual-blade tip. 

**Figure 2:**
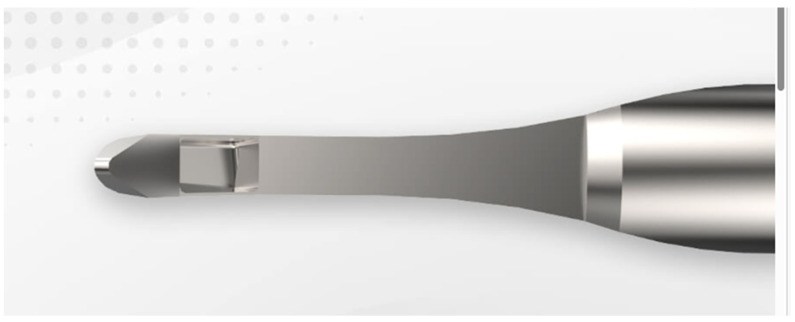
The Kahook dual bade is a device to excise the trabecular meshwork (internal trabeculectomy) between two parallel blades near the tip (product of New World Medical Rancho Cucamonga, CA).

Some have raised questions about the effectiveness of internal trabeculectomy [107]; however, numerous reports have documented the efficacy of Trabectome procedures [108,109,110,111,112,113,114,115,116,117,118,119,120,121,122,123,124,125,126,127,128,129,130,131,132,133,134,135,136,137,138,139,140,141,142,143,144,145,146,147,148,149] and Kahook dual blade (KDB) surgery [150,151,152,153,154,155,156,157,158,159,160,161,162,163,164,165,166,167,168,169,170,171,172,173,174,175,176,177,178,179,180] in maintaining the long-term control of the IOP (Figure 2).

### 5.3. Evolution of Internal Trabeculotomy, Which Opens Trabecular Meshwork without Excising Tissue

**Figure 3:**
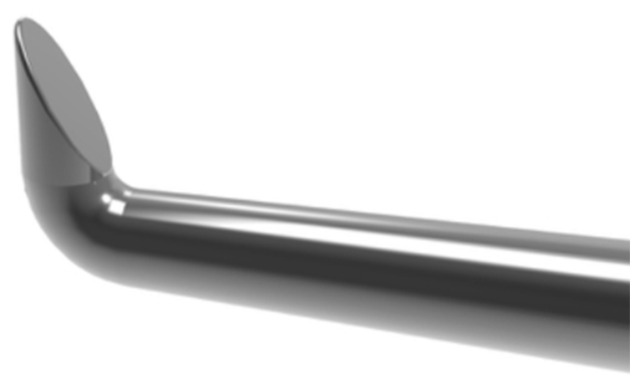
Tanito micro-hook for internal trabeculotomy: A device inserted into Schlemm’s canal to push away trabecular meshwork tissue (product of Inami, Tokyo).

The Tanito micro-hook (TMH, Figure 3) is a device for internal trabeculotomy that pushes away the trabecular meshwork to create an opening in Schlemm’s canal. The surgical outcomes of the TMH are comparable to those achieved with the Trabectome, KDB, and trabeculotomy ab externo [141,181,182,183,184,185,186,187,188,189,190,191,192,193] and it may surpass the efficacy of the iStent [194,195,196]. 

**Figure 4:**
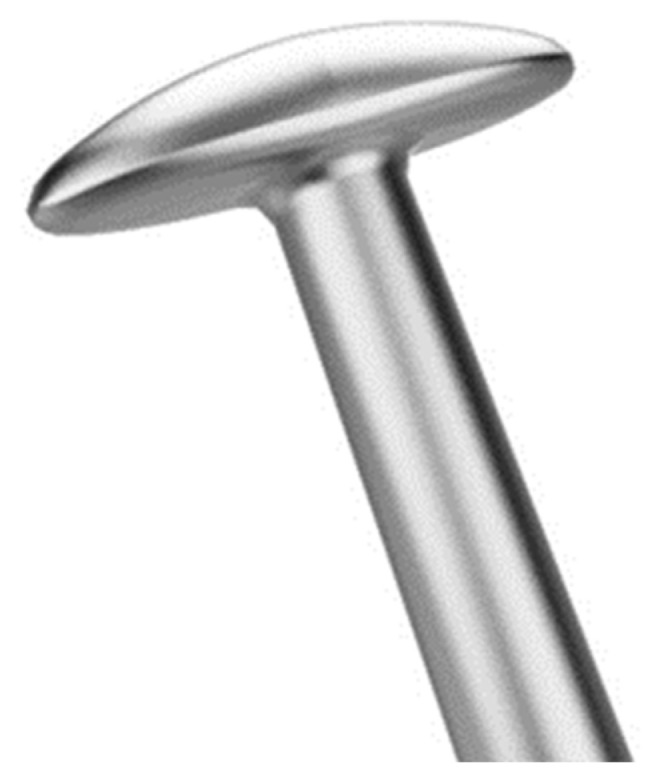
Chihara T-hook for internal trabeculotomy. The T-shaped head allows the bidirectional opening of the trabecular meshwork. The tip of this device is rounded and designed not to injure outer wall of Schlemm’s canal (product of Inami Tokyo; Handaya Tokyo; and AOI: Advanced Ophthalmic Innovations, Singapore).

The T-hook (Figure 4) is a novel device designed to permit the bidirectional opening of the trabecular meshwork without injuring the outer wall tissue of Schlemm’s canal [197]. 

Additionally, there are other new devices that have recently been invented to push away trabecular meshwork tissue [198].

### 5.4. Effects of Canal Expansion (Canaloplasty) without Opening the Trabecular Meshwork

Canaloplasty facilitates aqueous outflow by expanding Schlemm’s canal, and this technique has gained widespread adoption, primarily in Europe. The fundamental mechanism underlining IOP reduction in canaloplasty may be explained by four theories, one of which is the hinge–valve theory [199]. The tension of the conduits traversing Schlemm’s canal regulates both the aqueous inflow from the anterior chamber into Schlemm’s canal and the outflow from Schlemm’s canal to the collector channel. The proposed entry site for the aqueous chamber is a funnel-shaped canal inlet, and the regulator of the aqueous outlet is a hinged collagen flap. If Schlemm’s canal expands, the tension of the conduit increases, and the hinged collagen flap is pulled, opening the outlet valve. Another theory suggests that Schlemm’s canal ruptures under high pressure, resulting in multiple defects in the inner and outer walls of Schlemm’s canal [200,201]. The third theory involves the herniation of the inner wall of Schlemm’s canal into the collector channel. In other theories, this procedure effectively reduces the IOP by dilating the collapsed Schlemm’s canal, cleaning the collector channel, and stretching the trabecular meshwork. Even now, debate continues regarding the basic mechanism of IOP reduction following canal expansion. 

The technique involves the viscodilation of Schlemm’s canal, the insertion of a microcatheter (such as iTrack, OMNI, etc.), and the stretching of the trabecular meshwork using a 10-0 prolene thread, facilitating aqueous outflow. Additionally, the creation of an intrascleral lake may provide an alternative drainage route into the intrascleral and suprachoroidal space [202]. Planned goniopuncture can further enhance the outflow. In the case of failure following canaloplasty, trabeculotomy may be employed as the next step [203]. 

External trabeculectomy is more efficient than canaloplasty in reducing the IOP; however, the probability of success when using canaloplasty is not inferior to that of trabeculectomy, according to several meta-analysis studies [204,205,206].

In one report, conventional canaloplasty achieved better outcomes than viscocanalostomy [207]. There are numerous reports on the effects of canaloplasty [98,103,104,105,106,208,209,210,211,212,213,214,215,216,217,218,219,220,221,222,223,224,225,226,227,228,229,230,231,232,233,234,235,236,237,238,239,240,241,242,243,244,245,246,247,248,249,250,251,252,253,254,255,256,257,258,259,260,261,262,263,264]. Essentially, classic canaloplasty is performed externally and is not considered an MIGS procedure [203,208,209,210,211,212,213,214,215,216,217,218,219,220,221,222,223,224,226,227,228,229,230,231,235,236,239,241,242,245,249,250,255,256,261,262,263,264]. However, recent ab interno techniques using specific devices (such as iTrack, OMNI, etc.) are categorized as MIGS [98,103,104,105,106,225,232,233,234,237,238,240,243,244,246,247,248,251,252,253,254,255,256,257,258,259,260]. In the latest advancement of canaloplasty, Schlemm’s canal is “unroofed” [103,104,105,106,203,253,259,265]. If Schlemm’s canal is disrupted under high intracanal pressure [200,201], the difference between GATT or suture trabeculotomy and canaloplasty becomes unclear [265]. 

### 5.5. Streamline^®^ and Hydrus^®^

Streamline^®^ expands Schlemm’s canal using a viscoelastic material [266], while Hydrus, a Schlemm’s canal scaffold, combines elements of canaloplasty and the iStent to dilate Schlemm’s canal and establish a connection between the anterior chamber and Schlemm’s canal [260]. Both methods are considered to be techniques that are associated with canaloplasty. These reduce the IOP without significant complications. 

### 5.6. iStent

The iStent, a popular tool for canal surgery, offers a low risk of complications and a mild reduction in IOP. This is a type of stenting surgery that does not open the canal. There are many review papers that compare the outcomes between the iStent and canal opening MIGS [267,268,269,270,271,272]; therefore, it is not extensively reviewed in this study. 

### 5.7. Others

Viscocanalostomy is a technique that externally modulates Schlemm’s canal. It is not included in MIGS and not commented upon in this study [273]. Recently, viscocanalostomy has been largely replaced by canaloplasty [207]. High-frequency deep sclerectomy creates an intrascleral pocket internally and facilitates the uveo-scleral pathway, but it is not included in canal opening surgery [267].

## 6. Clinical Controversies Regarding Internal Canal Opening Surgery

### 6.1. Differences between Internal Canalectomy and Canalotomy

#### 6.1.1. Open or Closed? Does It Matter? 

When Minckler and colleagues introduced the “Trabectome”, the device was designed to create the permanent unroofing of the TM [25]. They believed that permanent unroofing was important for long-lasting IOP reduction, a concept that was later advanced by the Kahook dual blade (KDB) [274]. In contrast, trabeculotomy ab externo opens Schlemm’s canal through blunt trauma using a nylon filament or metal probe. This concept was succeeded by GATT and internal suture trabeculotomy, which creates a “shelf” in the angle. Similarly, new devices such as the Tanito micro-hook (TMH) and the T-hook push the TM tissue away and do not excise the TM, creating a double-door opening in the TM. 

When post-surgical wounds were studied using anterior segment OCT, the size of the wound was found to be greater in cases of internal trabeculectomy, such as that with the KDB, compared to internal trabeculotomy, such as that with the TMH. However, no significant difference in post-surgical IOP was noted [275]. 

From a clinical standpoint, no discernible difference in surgical outcomes was observed between the internal trabeculectomy cases, such as those involving the Trabectome and KDB, and the trabeculotomy cases, such as those involving trabeculotomy ab externo, GATT [83], the TMH [189], the T-hook [197], and 360-degree suture trabeculotomy [82,141,184,197,276,277,278,279,280]. 

This raises the question of whether the surgical outcomes after internal trabeculotomy differ from those after internal trabeculectomy.

However, there is still insufficient research to determine whether the long-term observations reveal distinctions between the two procedures [271,281]. The clinical findings and the histopathological studies conducted by Hamanaka and others [66] suggest that the issue regarding whether Schlemm’s canal is opened or closed may not be the critical factor; instead, the sealing of the inner surface of the canal is the issue. 

On the other hand, there is a debate regarding whether the meridional extent of the canal opening affects the surgical outcomes (see Section 6.2). Some believe that a larger opening is beneficial for a greater IOP reduction [101,282,283,284], while others hold the contrary view. 

#### 6.1.2. Comparison between Conventional Trabeculectomy and MIGS

Meta-analysis is a desirable approach for the evaluation of the efficacy of new surgical techniques; however, the number of randomized clinical trials in the area of MIGS is not sufficient [285]. 

Several reports indicate that ab interno trabeculotomy provides a similar IOP reduction to trabeculectomy, with a lower risk of complications [128,286]. The post-surgical IOP outcomes for the Ahmed glaucoma valve and Baerveldt implant may be comparable [121,123]. However, others suggest that conventional trabeculectomy has greater efficacy in reducing the IOP compared to internal trabeculotomy [287]. Most reports agree that trabeculectomy also carries a higher risk of complications [288]. 

### 6.2. Does the Extent of the Canal Opening Area Impact the Surgical Outcomes?

In a clinical study, the area of active circumferential flow in Schlemm’s canal was restricted. Fellman and Grover reported that downstream episcleral vein bleaching is confined to two clock hours from the edge of the Trabectome incision. In their report, the bleaching of the episcleral veins was shown to occur within 41 degrees infero-nasally and 13 degrees supero-nasally from the incisional end using the Trabectome [289]. This may explain why the effects of the iStent on the IOP are inferior to those of other internal trabeculotomy techniques [130,167,195,290,291,292,293]. In a meta-analysis study, the outcome of the iStent was inferior to that of the Hydrus [271]. 

Ellingsen and Morton found that the aqueous outflow was better when a wide area of the trabecular meshwork was opened [294]. Rosenquist and others confirmed the better IOP reduction with a wider Schlemm’s canal opening in cadaver eyes [295]. Several clinical reports also suggest that the wider opening of the canal leads to a better IOP reduction [97,101,282,296,297].

However, when the canal opening extends beyond 90 degrees, the effects of widening the canal opening on the post-surgical intraocular pressure do not exhibit a consistent trend. Several studies indicate that there is no significant difference in the extent of IOP reduction when the canal opening exceeds 90 degrees [182,298,299,300,301,302,303]. In a hypothetical model, the decrease in outflow resistance was greater in eyes with a 4 h incision than those with a 1 h incision. However, the effects of expanding the incision on the post-surgical IOP reached a saturation point when the excision area exceeded 90 degrees [304]. In eyes where the canal opening is expanded to 360 degrees, there is no further decrease in outflow resistance [304]. The wider the canal opening, the greater the intracameral bleeding and obstruction of collector channels [305].

It has been reported that only one or two active aqueous veins are enough to drain a sufficient amount of aqueous humor to maintain a normal IOP [77,78]. Thus, the survival of only a few active collector channels may be sufficient to sustain normal intraocular pressure. After the canal opening surgery, the gradual coverage of the collector channel opening due to the elongation of the Schlemm’s canal endothelium, along with the proliferation of fibrous tissue, constitutes a slowly progressing phenomenon and may lead to a gradual elevation in IOP [66]. It may require a long time for the complete obstruction of 80 collector channels to occur. 

If the collector channel at the site of the canal opening is intact, a localized opening may be sufficient to obtain clinical success; however, it is difficult to estimate the clinical integrity of the intra-scleral outflow before surgery. If the outflow resistance is abnormal in a specific meridian, the opening of Schlemm’s canal in the affected area may not yield a good outcome. From this perspective, “complete trabeculotomy” (360-degree canal opening) may offer an advantage in achieving constant results in eyes with a localized impairment in the outflow system. When comparing the surgical outcomes between complete trabeculotomy (360-degree canal opening) and “partial trabeculotomy” (less than 360-degree canal opening), several reports demonstrate better outcomes with complete trabeculotomy [97,101,296,297], although others do not [299,300,303]. 

It is still unclear whether a wide canal opening is beneficial to the restoration of the active aqueous vein or not. A prospective and long-term follow-up study is needed to address this question. 

### 6.3. Does the Type of Glaucoma Affect Canal Opening Surgery Outcomes?

#### 6.3.1. Steroid-Induced Glaucoma 

In steroid-induced glaucoma, an abnormal extracellular matrix (ECM), consisting of abnormal collagen and dense homogenous material, accumulates adjacent to the inner wall of Schlemm’s canal. These ECM characteristics are different from those observed in POAG [306]. Cellular proliferation and migration in the sclera are restricted, while the cell size may be increased after steroid administration [307]. Clinically, Schlemm’s canal and CC are generally unaffected by steroids, and a favorable response to the canal opening surgery is expected. Many reports indicate positive outcomes following internal trabeculotomy or internal trabeculectomy [114,181,245,261,284,308,309,310,311]. Therefore, steroid-induced glaucoma may be considered a suitable candidate for internal canal surgeries.

#### 6.3.2. Secondary Glaucoma: Uveitic Glaucoma 

When inflammatory cells obstruct the trabecular meshwork, the intraocular pressure increases. In the case of prolonged inflammation, scar tissue is formed, which impairs the aqueous outflow. In canal surgery, post-surgical inflammation may promote the post-surgical proliferation of fibrous tissue and induce the elongation of the SCE [181,308]. 

However, when the inflammation was mitigated and the post-Schlemm’s canal route was preserved, positive outcomes were reported in canal surgeries for secondary glaucoma [185,312,313]. Some cases of uveitic glaucoma may respond similarly to POAG or steroid-induced glaucoma [100,284]. 

#### 6.3.3. Pseudoexfoliation (Exfoliation) Glaucoma

In contrast to POAG, (pseudo-)exfoliation glaucoma (PEG) is characterized by a higher IOP, faster visual field deterioration, a poor response to medical therapy, and an increased need for surgical intervention. While some reports demonstrate positive outcomes with canal surgeries in PEG cases [17,52,55], others do not. 

The reduction in IOP through canal surgery in PEG cases is significant [17,52,102,115,139,314,315], often surpassing that observed in POAG [55,316], or an equivalent outcome between PEG and POAG is shown [94,119,317]. However, it is noteworthy that the pre-surgical IOP is typically higher in PEG cases, which may contribute to a greater IOP reduction following canal surgery. This substantial IOP reduction in PEG cases may simply reflect the inherent nature of canal surgery, which tends to achieve a higher IOP reduction in cases with a high preoperative IOP. Consequently, the final IOP in PEG might remain high, potentially leading to a poor final outcome [181]. In addition to the final IOP, the fluctuation of the IOP level may cause nerve damage. Therefore, the efficacy of canal surgery in treating PEG remains unclear. 

### 6.4. How High Can the Preoperative Intraocular Pressure Be to Indicate Canal Opening Surgery?

In many reports, the surgical reduction in the IOP in canal surgery is more pronounced in eyes with a higher pre-surgical IOP [181,183,192,318,319,320]. Tanito reported the following association: (percentile IOP reduction) = 2.1 × (preoperative IOP) − 15.7 [183]. A clear correlation exists between the pre-surgical IOP and the extent of IOP reduction [183]. We reported a marginal association between the pre-surgical IOP and post-surgical IOP in phaco-trabeculotomy cases, reporting the following regression equation: (post-surgical IOP) = 12.812 + 0.166 × (pre-surgical IOP), *p* = 0.0629. In this study, the critical pre-surgical IOP required to achieve 18 mmHg was 31.3 mmHg [319]. However, the association between the pre- and post-surgical IOP was weak and it may not follow a linear regression line; thus, it requires further elucidation. The upper limit of the pre-surgical IOP for consideration of canal opening surgery has not yet been determined.

### 6.5. Do Pre- and Post-Surgical Eye Drops Affect Outcomes?

The use of certain drugs may influence or alter the surgical outcome. A parasympathomimetic drug is employed to prevent post-surgical peripheral anterior synechia (PAS) formation, although its effects have not been comprehensively assessed [321]. It should be noted that parasympathomimetic drugs may induce post-surgical inflammation, potentially leading to PAS formation.

The application of a vasoconstrictive agent may potentially limit intracameral bleeding [322]. The use of Rho-associated coiled-coil-containing protein kinase (ROCK) inhibitors may modulate the contractile elements of the collector channel and positively impact the post-surgical IOP. However, the available data are limited, and additional studies are needed to validate these findings [323,324]. The response to ROCK inhibitors may be useful in predicting the outcome of MIGS, while the prolonged or multiple use of anti-glaucoma medications may adversely affect the outcome [325,326,327]. 

### 6.6. Does the Combination of Cataract Surgery Benefit the Outcomes of Canal Opening Surgery?

The extraction of the lens results in the retro-positioning of the iris diaphragm and increases the tension on the trabecular meshwork [199]. Simple lensectomy widens the angle and intensifies the tension of the trabecular meshwork, consequently reducing the IOP [328,329,330,331]. In angle-closure glaucoma, lens extraction has a favorable impact on the post-surgical IOP [137,329]. 

On the other hand, the results of a meta-analysis of the outcomes of standalone Trabectome (39% reduction) and a combined phacoemulsification–Trabectome (27% reduction) were not conclusive [332]. Regarding POAG cases, there is ongoing debate concerning the effects of concomitant cataract surgery and canal surgery in terms of their impacts on the post-surgical IOP. While several studies indicate positive effects [97,314,315,320,333,334,335,336,337], conflicting findings are reported by others [83,93,303,338,339,340,341,342].

It is worth noting that combined cataract surgery negatively affects the outcomes of trabeculectomy; however, this is not the case in canal surgeries [102].

### 6.7. How Does a Post-Surgical IOP Spike and Intracameral Bleeding Affect MIGS Outcomes?

Here, we must be careful to note the difference between simple hyphema and clot formation. Surgical trauma to vascular tissue triggers the release of von Willebrand factor, initiating a cascade of events from platelet adherence and the conversion of fibrinogen to fibrin, which leads to peripheral anterior synechia (PAS) formation and the potential occlusion of the collector channel orifice [92,343]. Rao reports that the development of PAS is associated with a spike in the IOP [92]. 

Even though the trabecular meshwork tissue is rich in tissue plasminogen activator [344] and facilitates the dissolution of a blood clot to enhance aqueous humor drainage, once a clot is formed, it may worsen the tissue adhesion. Ishida and colleagues have reported a close correlation between clot formation and a subsequent elevation in the post-surgical IOP [196]. Shi, Quan, and others have emphasized that a spike in the IOP constitutes a risk factor for poor IOP control [89,90,91]. In other reports, clot formation is highlighted as a significant risk factor for re-operation [305] and may lead to a gradual increase in the post-surgical IOP [79]. 

### 6.8. Does Prior Selective Laser Trabeculoplasty (SLT) or LTP Affect MIGS Outcomes?

A modest reduction in IOP is achieved with selective laser trabeculoplasty, without any significant complications. This may not affect the surgical outcome of the canal surgery [345,346]; however, others report adverse effects of SLT [148,336,347]. After a failed SLT, canal opening surgery is a viable candidate option to reduce the IOP [348,349,350]. However, caution is necessary, as there is a report suggesting that SLT may not be useful as a subsequent step following failed canal surgery [351]. 

### 6.9. What Are the Risk Factors for the Failure of Canal Surgery?

As mentioned previously, uveitic glaucoma and pseudoexfoliation glaucoma, a history of SLT and LTP [347], and post-surgical PAS or spikes are risk factors for failure. Neovascular glaucoma is contraindicated for canal surgery, with the exception of one report [88]. 

When the preoperative IOP is high, the decline in IOP is large; however, the final IOP value is often high, so a high preoperative IOP is a risk factor for failure [181,320,352,353]. Regarding traumatic glaucoma, we do not have enough evidence, and it is therefore difficult to comment on. A younger age [87,336,354] was shown to be a risk factor for failure, except in one report [86]. This finding may contrast with the findings of older studies, in which the outcome of trabeculotomy ab externo was better in congenital glaucoma than in juvenile-onset glaucoma [31,32]. 

A high central corneal thickness [353,354] and myopia [355,356,357] were listed as risk factors, except in one study [358]. Being male may affect the outcome [359]. It is noteworthy that better outcomes are reported in Hispanic individuals [315]. However, the Black ethnicity was associated with a higher risk of reoperation [320].

### 6.10. Risk of Postoperative Ciliary Effusion

Canal-based MIGS procedures do not typically result in severe post-surgical hypotension and are generally considered safe. Ciliary effusion is a rare complication, although it may occasionally be observed [187,360,361,362]. This complication can be triggered by inadvertent damage to the pectinate ligament and the formation of a ciliary cleft. Precise opening of the trabecular meshwork is crucial in minimizing the risk of post-surgical ciliary effusion.

## 7. Conclusions

This review explores the history of canal opening surgery, the several types of procedures available, the clinical controversies surrounding them, and the risk factors associated with their failure. 

External trabeculotomy, since its inception, has faced numerous challenges and has not gained global acceptance for the treatment of adult-onset POAG. It was later modified into internal trabeculotomy, which has recently become more popular. Initial experiments on normal monkey eyes indicated the excessive regeneration of the trabecular meshwork, casting doubt on the efficacy of external trabeculotomy and hindering its widespread adoption in clinical practice. However, more recent studies showing mild histopathological tissue responses in humans, combined with numerous clinical success stories, have led to a shift in perspective. Currently, canal opening surgeries, classified as minimally invasive glaucoma surgeries (MIGS), have gained popularity. Nevertheless, distinctions from other procedures, such as canaloplasty and stenting surgeries, have sparked significant debate. Additionally, there remains a debate concerning the surgical outcomes based on the surgical method, type of glaucoma, preoperative intraocular pressure levels, effects of concurrent cataract surgeries, impacts of postoperative anterior chamber hemorrhage, effects of treatments such as SLT, and risk factors associated with the surgery.

**Future Directions:** Canal opening surgery currently offers good postoperative visual acuity and an effective IOP reduction, making it a promising treatment for mild to moderate glaucoma. While there are several surgical methods and devices available, they are being improved annually and should be explored to ensure that they become more cost-effective and user-friendly in the future.
